# Validity of leg length measurement in the supine and standing position compared with pelvic survey X-ray after total hip arthroplasty

**DOI:** 10.1007/s00402-023-05014-6

**Published:** 2023-08-02

**Authors:** Steffen Brodt, Marcel Schulze, Benjamin Jacob, Georgi Wassilew, Dimitri Nowack, Sebastian Rohe, Georg Matziolis

**Affiliations:** 1 Department of Orthopedics, Jena University Hospital, Campus Eisenberg, Klosterlausnitzer Str. 81, 07607 Eisenberg, Germany; 2 Center for Orthopaedics, Trauma Surgery and Rehabilitation Medicine, University Medicine Greifswald, Ferdinand-Sauerbruch-Straße, 17475 Greifswald, Germany

**Keywords:** Total hip arthroplasty, Leg length difference, Limb length measurement, Technique

## Abstract

**Introduction:**

The correct adjustment of leg length is a major goal in the implantation of total hip replacements (THRs). Differences in leg length can lead to functional impairment and patient dissatisfaction. By determining leg length at an early stage, before the patient is discharged from hospital, compensatory measures such as the production of special insoles or orthopaedic footwear can be initiated promptly if there is a difference in leg length. Due to shortening of the period of time spent in hospital, the traditional measurement of leg length in a standing position may be increasingly subject to error. A protective posture immediately after surgery or the presence of a twisted pelvis, for example, due to scoliotic spinal misalignments, falsifies the measurement result in the standing position. Here, the measurement of leg length in the supine position may prove to be accurate immediately postoperatively, regardless of potential sources of error, and is to be compared with measurement in the standing position versus radiological measurement on the AP pelvic survey.

**Material and methods:**

The present retrospective study included 190 patients who had undergone primary total hip arthroplasty. The leg length difference (LLD) of the patients was determined pre- and postoperatively both in the supine and standing position and compared with the postoperative radiological pelvic survey image.

**Results:**

Postoperatively, it was shown that the mean length measured was 0.35 mm too long in the supine position and 0.68 mm too short in the standing position (*p* value < 0.001). Determination of the average absolute measurement error produces a deviation of 4.06 mm in the standing and 4.51 mm in the supine position (*p* value 0.126).

**Conclusions:**

It is shown that the postoperative measurement of LLD in the supine and standing position is equally valid and sufficiently accurate, compared with the gold standard of measurement on a radiograph.

## Introduction

When implanting a total hip replacement (THR), the aim is to reconstruct the anatomy and thus the biomechanics of the hip joint in an ideal way. This includes the correct reconstruction of the centre of rotation (COR) on the acetabular side and the restoration of leg length, offset and torsion on the femoral side. Aids such as navigation or intraoperative X-ray are available for the exact positioning of implants [1–3]. However, on one hand, very few surgeons make use of these aids. On the other, even navigation and intraoperative X-rays do not guarantee perfect implant positioning. One of the most common side effects after THR implantation is the intraoperative change in leg length, which usually leads to a lengthening of the operated side [4–6].

The measured and the patient’s subjectively perceived leg length are not always congruent, especially in the immediate postoperative phase.

In a retrospective study of 1114 patients, 329 (30%) showed a subjectively perceived leg length difference. Of these, however, a leg length difference could only be confirmed radiologically in 36%. Nevertheless, a subjectively perceived leg length discrepancy was associated with a lower Oxford Hip Score (OHS) [7].

The radiological measurement of leg length is carried out on the basis of an AP pelvic image by placing a line through Köhler’s teardrop figure and then moving this parallel to the lesser trochanter [8, 9]. However, some hospitals do without a pelvic survey image for reasons of radiation protection and only carry out a hip AP and lateral view postoperatively. Some authors even question the need for routine postoperative radiographs after hip arthroplasty [10].

Clinical measurement is performed with the patient in the standing position. Leg length discrepancy blocks are placed under the shorter limb in 5 mm increments until the iliac crests are parallel and the patient is standing straight [11].

A third option is to measure the actual leg length using a tape measure from the anterior superior iliac spine to the lateral malleolus on the supine patient [11].

Various comparisons of different methods and an assessment of their validity can be found in the literature [12, 13]. In a recent meta-analysis, a wide variance in the reported intra- and inter-rater reliability was found for most measurement techniques [14].

The routine determination of leg length prior to hospital discharge is important to the extent that differences can be immediately compensated for by orthopaedic devices such as orthotics to avoid negative influences on the musculoskeletal system [15, 16].

With hospital stays becoming shorter and shorter, at the time of discharge patients often still reflexively protect the operated leg and hold it in a slight flexion posture. This can falsify the measurement of LLD in the standing position. In contrast to the standing position, the operated leg is not loaded when lying down, so that this potential measurement error should not occur. Other factors that lead to an active compensatory pelvic obliquity (e.g. decompensated degenerative lumbar scoliosis) should also have at least less of an effect in the supine than in the standing position [17, 18].

The question therefore arises whether the measurement of the malleolar distance on the supine patient is more accurate than the traditional determination of LLD in the standing position. The quantification of LLD on the pelvic survey image is taken as the gold standard.

## Methods

This study was approved by the ethics committee of our university hospital with the number 2023-2875-Daten and registered in the German Register of Clinical Trials (DRKS) with the number DRKS00031109. 190 patients who had undergone total hip arthroplasty (THA) in a tertiary hospital between September 2019 and October 2021 were investigated.

**Fig. 1:**
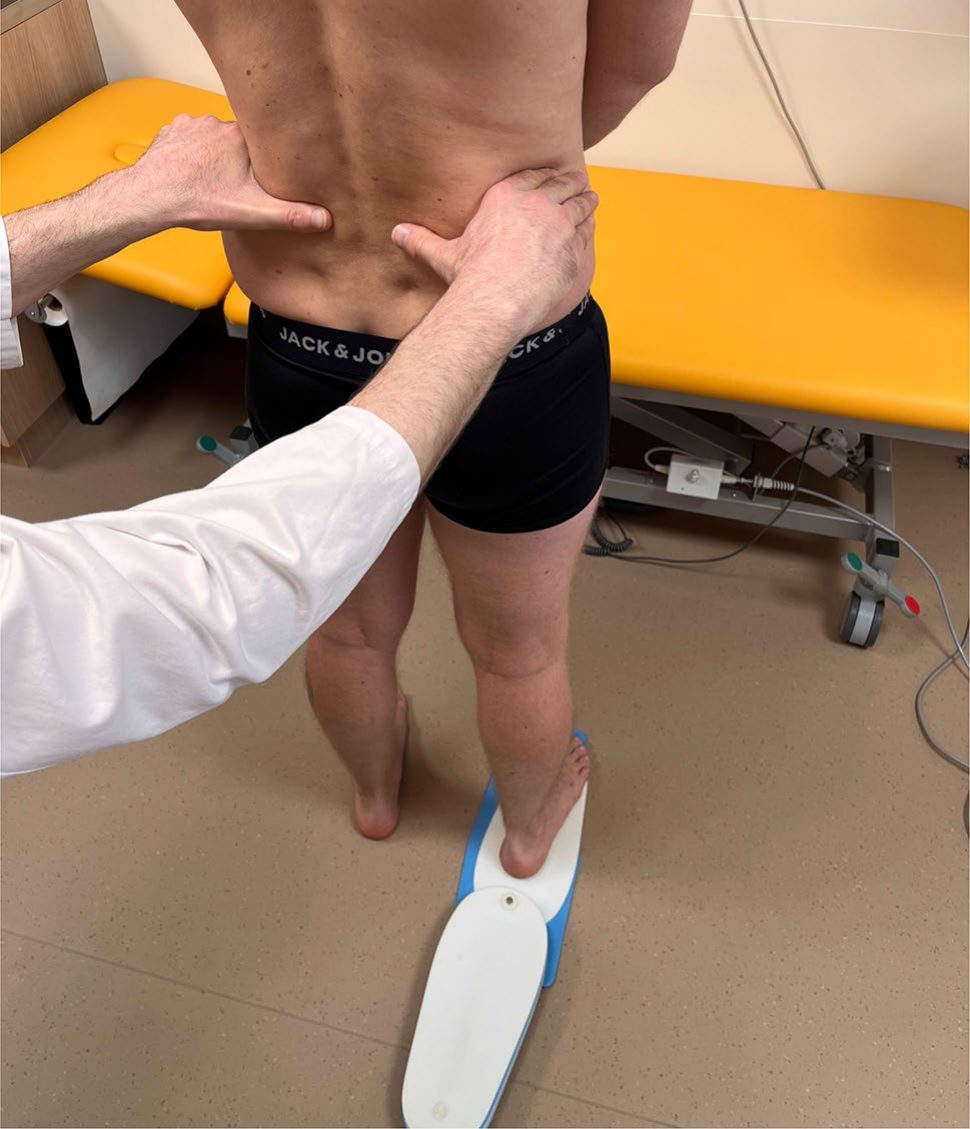
Leg length measurement in the standing position by blocks under the right foot up to the horizontal pelvic position

The differences in leg length were measured one day preoperatively in the standing position by inserting leg length discrepancy blocks up to the horizontal pelvic position (Fig. 1).

The patient stood with the knees extended and the legs shoulder-width apart. The iliac crest was palpated. The leg discrepancy was balanced until the iliac crests were parallel to the floor.

**Fig. 2:**
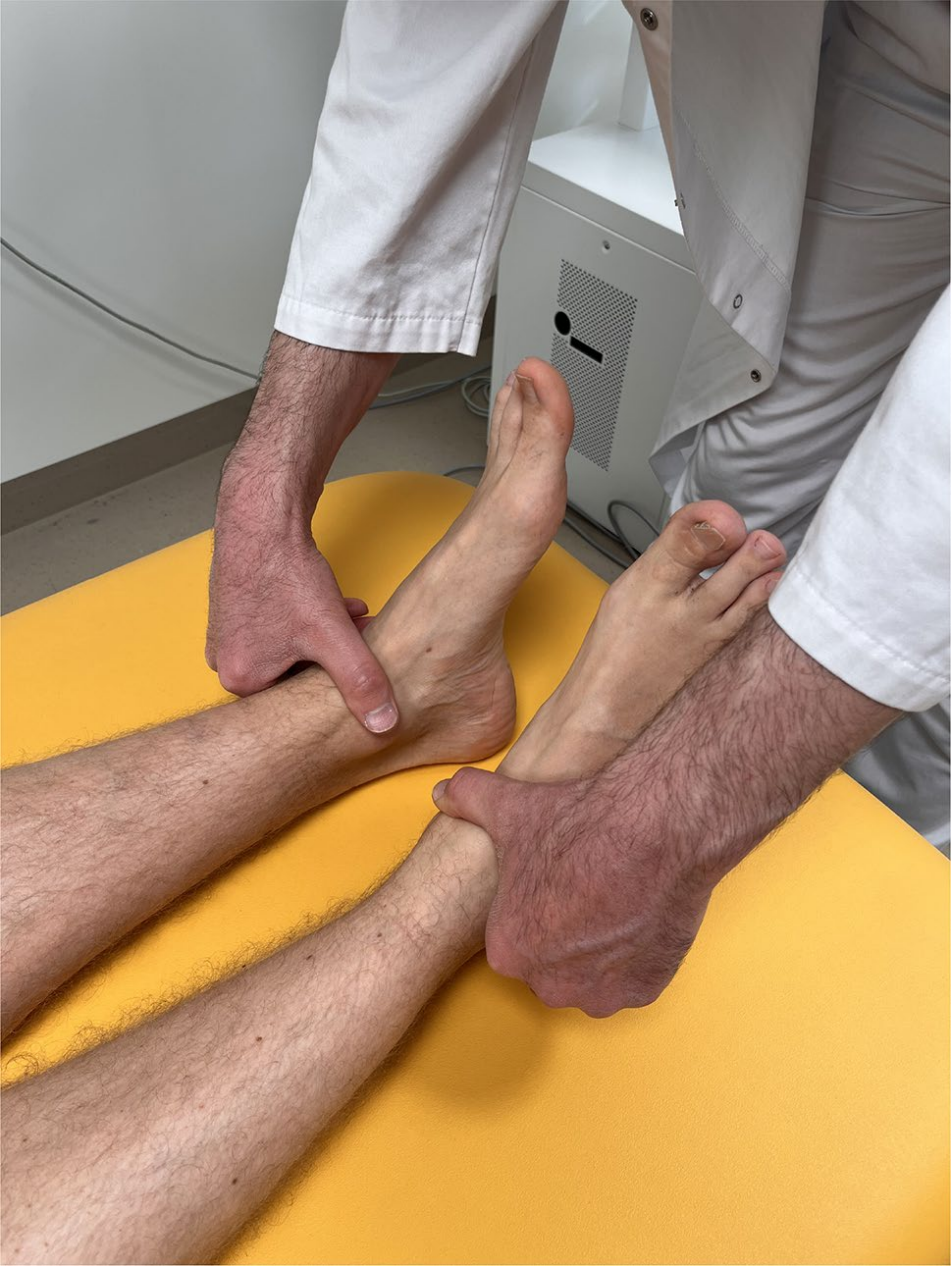
Leg length measurement in the lying position with the malleoli placed against each other

In the supine position, the patient was measured lying flat and straight in their hospital bed with the malleoli placed against each other (Fig. 2). The difference in length between the malleoli was determined [19]. The Weber-Barstow manoeuvre was applied to relax the pelvic muscle and the lower limb [20]. These two measurements were generally performed by the attending surgeon. The measurement was taken with a resolution of 5 mm. Additionally, patients were asked to state their subjectively perceived difference in leg length.

**Fig. 3:**
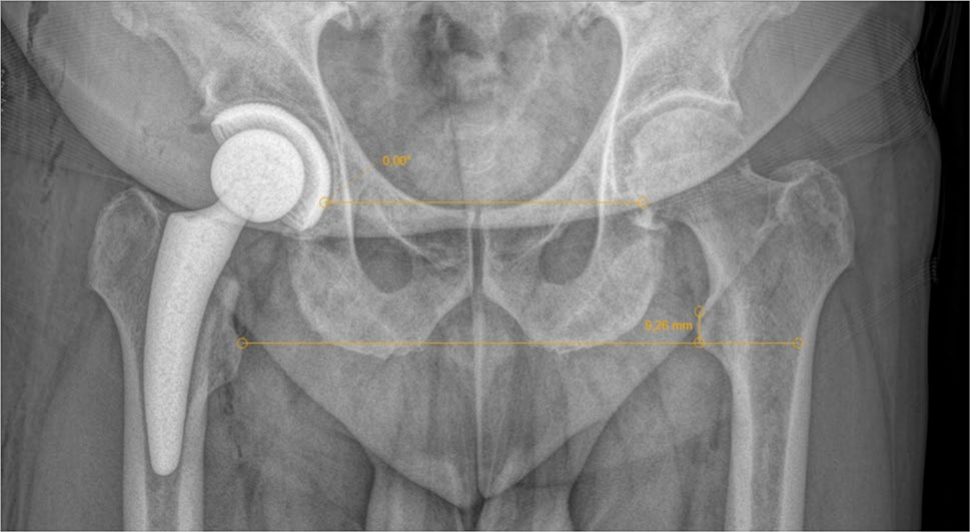
Postoperative measurement on a pelvic survey X-ray image

Independently of this and without knowledge of the clinically measured values, a single investigator took measurements on the standard preoperative and postoperative pelvic survey X-ray images. The standard radiographs were taken in the standing position. For this purpose, a line was drawn through Köhler’s teardrop figure and this was moved parallel to the lesser trochanter (Fig. 3).

Here, the difference between the two legs could now be determined, taking into account the radiological magnification factor. The measurement achieves a resolution and accuracy of 1 mm [21].

Depending on the surgeon, surgery was performed in the supine or lateral position using a posterolateral, transgluteal or anterolateral approach.

On the 4th postoperative day, standard measurement of leg length was performed in the standing and supine position by the ward physician. In addition, patients were again asked to state their subjective perception. Furthermore, on the same day, the standard X-ray control was carried out by means of a pelvic survey image in the standing position. The measurement of the patient in the supine position was compared with the X-ray image and the measurement of the patient in the standing position was compared with the X-ray image. All patients were followed up in our outpatient clinic 3 months postoperatively. The leg length was measured again in the standing and supine positions and the patients were asked to state their subjectively perceived leg length.

The data were analysed statistically using SPSS (IBM SPSS Statistics 25, New York, USA). The data were first tested for normal distribution using the Kolmogorov–Smirnov and Shapiro–Wilk tests. Group differences were tested for using Wilcoxon's test for paired samples at a level of significance of 0.05.

Subsequently, the mean deviation of the LLD measured in the supine and standing position from the radiologically determined LLD and the respective mean absolute error were determined. In addition, the intraclass correlation coefficient (ICC) and Cronbach’s alpha of the supine and standing measurements were calculated compared to the gold standard of the pelvic survey.

## Results

**Fig. 4:**
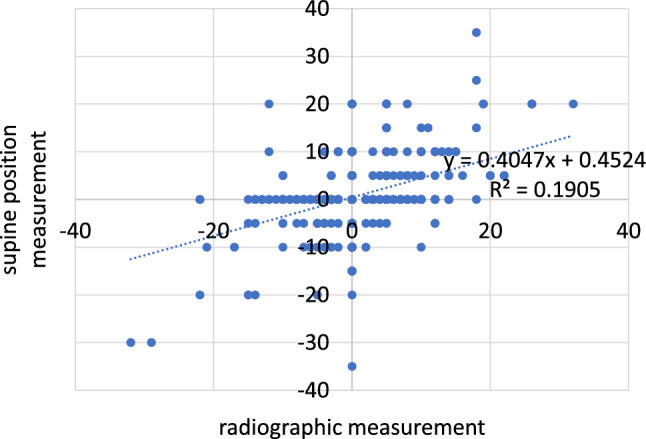
Supine position measurement versus radiographic measurement

**Fig. 5:**
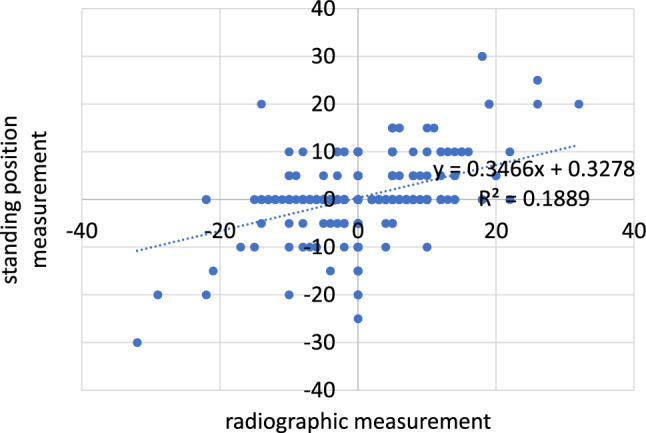
Standing position measurement versus radiographic measurement

A total of 190 patients were included in this study. Among them were 93 men and 97 women. 95 patients underwent surgery on the right side. 90 patients received a hip replacement on the left side and 5 patients underwent simultaneous bilateral surgery. The patients were between 22 and 86 years old (Figs. 4 and 5).

Preoperatively, the measurement in the standing position was 1.1 mm (SD 7.0 mm) longer and the measurement in the supine position 0.7 mm (SD 7.4 mm) longer than in the radiograph (*p* = 0.493) (Figs. 1 and 2). The absolute measurement error was 4.5 mm (SD 5.4 mm) in the standing and 4.9 mm (SD 5.6 mm) in the supine position (*p* = 0.191).

Postoperatively, the measurement was found to be on average 0.4 mm (SD 6.5 mm) too long in the supine position and 0.7 mm (SD 6.1 mm) too short in the standing position (*p* < 0.001). The absolute measurement errors were 4.1 mm (SD 4.6 mm) in the standing position and 4.5 mm (SD 4.6 mm) in the supine position (*p* = 0.126). Both pre- and postoperatively, the absolute error was thus less than 5 mm for the standing and supine measurements alike. A superiority of one clinical measurement method over the other was not found.

Furthermore, the test reliability of the clinical leg length determination in the standing and supine positions compared with measurement on the X-ray image was tested by means of Cronbach’s alpha and the intraclass correlation coefficient (ICC). The values achieved in Cronbach’s alpha and ICC indicate poor reliability of the measurements in the standing and supine positions (Tables 1, 2, 3, 4).

**Table 1:** Cronbach’s alpha of leg length measurement in the supine and standing position versus X-ray

Cronbach’s α	Pre-OP	Post-OP	Follow-up
Supine position	0.605	0.604	0.506
Standing position	0.597	0.608	0.503

**Table 2:** Interpretation of Cronbach’s alpha

Cronbach’s α	Interpretation
> 0.7	Acceptable
> 0.6	Questionable
> 0.5	Poor/low
< 0.5	Unacceptable

**Table 3:** Intraclass correlation coefficient of leg length measurement in the supine and standing position versus X-ray

Intraclass coefficient (ICC)	Pre-OP	Post-OP	Follow-up
Supine position	0.433	0.432	0.338
Standing position	0.425	0.437	0.336

**Table 4:** Interpretation of the intraclass correlation coefficient (ICC)

Intraclass correlation coefficient (ICC)	Interpretation
0.00–0.40	Poor
0.40–0.75	Fair to good
0.75–1.0	Excellent

The leg length difference perceived by the patients was compared with the leg length difference measured in the radiograph (true LLD) (Table 5). At a radiologically measured difference of 5 mm, only about a quarter of the patients expressed the subjective feeling that they had a leg length difference, and at 10 mm about a third. A measured leg length difference of 15 mm was perceived by 7 out of 9 patients (78%) immediately after surgery and by 5 out of 9 patients (56%) at follow-up (Table 5).

**Table 5:** Patients perceived leg length difference (LLD) versus true LLD

True LLD	Pre-OP patients	Pre-OP perceived	Post-op patients	Post-OP perceived	Follow-up patients	Follow-up perceived
5 mm	62	14 (22%)	80	20 (25%)	80	18 (23%)
10 mm	27	9 (33.3%)	34	12 (35%)	34	11 (32%)
15 mm	8	6 (75%)	9	7 (78%)	9	5 (56%)

## Discussion

The main result of the present study is that the determination of LLD in the supine and standing positions are equally good and, with a mean error of less than 5 mm, sufficiently accurate.

However, reliability showed only moderate results, without differences between the measurement methods in the supine or standing position. A systematic error, i.e. a consistently too long or too short measurement with a specific method, could be excluded.

This would make it possible to avoid a difficult mobilisation of the patient and save time by measuring their leg length in bed, for example, during the morning ward round. Measures can then be taken immediately to address any differences in length leg found. If patients can only be measured after sufficient mobilisation and in an appropriate upright position, there is often too little time to organise a shoe adjustment before discharge.

In addition, patients who, due to the course of the operation, have to take weight off their operated leg for a prolonged period of time and therefore have a protective posture when standing can thus be measured more accurately.

Furthermore, it can be stated that the leg length measured on the X-ray image differs from the perceived leg length in the majority of cases. Only at a leg length difference of 15 mm and above is this indeed perceived by the majority of patients.

In the AP pelvic survey image, only the differences in length between the lesser or greater trochanters are considered [21]. A leg length discrepancy due to laterally different growth of the femur or tibia in length thus leads to incorrect determination of LLD with this method.

The accuracy of LLD determination in both the standing and supine positions is limited by the soft tissue mantle around the anterior superior iliac spine and the iliac crest, as both clinical methods require correct palpation of these landmarks. As different studies have shown, this error is as high as 4.6 mm, even in very experienced examiners [22, 23].

Because there is no difference between measurement of LLD in the supine and the standing patient in reference to measurement on the radiograph, in our opinion the postoperative clinical measurement of LLD can be performed both in the supine and standing positions.

## Data Availability

The data that support the findings of this study are not openly available due to reasons of sensitivity and are available from the corresponding author upon reasonable request.
